# SENTINEL-DL: a forensic framework for device attribution using motion sensor data

**DOI:** 10.1038/s41598-025-34734-5

**Published:** 2026-01-29

**Authors:** Attaullah Buriro, Abdul Baseer Buriro, Tahir Ahmad, Flaminia Luccio, Muhammad Azfar Yaqub, Markus Zanker

**Affiliations:** 1 School of Computer Science and Electronic Engineering, University of Essex, Colchester, UK; 2 Department of Electrical Engineering, Sukkur IBA University, Sukkur, Pakistan; 3 Security and Trust Center, Fondazione Bruno Kessler (FBK), Trento, Italy; 4 Department of Environmental Sciences, Informatics and Statistics, Ca’ Foscari University of Venice, Venice, Italy; 5 Faculty of Engineering, Free University of Bolzano, Bolzano, Italy; 6 University of Klagenfurt, Klagenfurt, Austria

**Keywords:** Electrical and electronic engineering, Computer science, Information technology

## Abstract

This paper introduces SENTINEL-DL-a novel forensic framework which leverages accelerometer sensory data to associate motion-based digital evidence to its corresponding smartphone or smartwatch models. SENTINEL-DL analyzes robust tamper-resistant intrinsic motion signatures (profiled using built-in 3D accelerometers) to establish device associations. Technically speaking, it leverages small differences in linear acceleration to identify and associate the readings with its generating device. SENTINEL-DL utilizes machine learning models including random forest (RF), deep neural networks (DNN) and convolutional neural networks (CNN) to drive its association during the matching process, i.e., unknown sensory data against a reference database containing device profiles from known sources. The results of empirical tests show that SENTINEL-DL for smartphones and smartwatches, respectively, achieves a true positive rate (TPR) of 93.99% and 92.65%, a false acceptance rate (FAR) of 0.66% and 1.22%, and an overall accuracy of 98.76% and 98.97%. SENTINEL-DL being light-weight promises investigators a dependable analysis solution for motion sensor evidence while providing digital fingerprinting capabilities and forensic authentication support. The research demonstrates how motion sensor data can be utilized in digital forensic investigations to develop improved device fingerprinting and forensic verification methodologies.

## Introduction

The proliferation of smart devices, i.e., smartphones and smartwatches, has transformed digital ecosystems. Beyond their traditional role in communication, they are now widely used in various domains, including health care^1,2^, activity recognition^3,4^, users’ attribute estimation^5,6^ and user authentication^7,8^. These devices produce and collect large quantities of sensory data and share this data with networked systems. Needless to say, this connectivity improves functionality and user convenience, however, it also introduces significant challenges related to investigations, particularly in digital evidence associations, device authentication, and other security concerns^9–11^.

In forensic investigations, the most essential task is to perform correct device-incident association for crimes including cybercrime, fraud and unauthorized network access^12^. Traditional methods of device-incident associations, i.e., through Media Access Control (MAC address—The MAC address is a unique identifier assigned to network interfaces for communication within a physical network. While it serves as a fundamental identifier, MAC addresses can be altered through software tools or system modifications, particularly on devices with elevated privileges (e.g., rooted or jailbroken systems)) and cryptographic key-based associations^13,14^ have shown to be vulnerable to spoofing^15^, hardware tampering^16^ and replay attacks^17^. MAC address spoofing enables attackers to mask a device’s true identity, while cryptographic key-based techniques can facilitate unauthorized access to otherwise secure systems. Technically speaking, if an attacker obtains these keys, they can decrypt sensitive information, impersonate legitimate users, or alter data without detection. Such compromises often result from vulnerabilities in key management practices, weak encryption algorithms, or inadequate protection of key storage. Furthermore, forensic investigations frequently encounter cases where traditional device identifiers, i.e., device logs, or network-based records, are either absent of compromised, necessitating alternative approaches for reliable evidence-device attribution. To this end, there is an increasing requirement for stronger, passive, and non-intrusive methods of evidence-device attribution.

The process of associating the sensory readings to its source (similar to associate a captured image to a camera) is a critical area of study in mobile forensic due to its significance for several reasons: However, this enables device identification by exploiting the subtle variations in these readings caused by hardware variations, manufacturing tolerances, and software configurations. These “sensor fingerprints” are vital for linking criminal activity to a suspect device^18,19^. Secondly, this data could also be used in reconstructing events during criminal investigations or disputes. For example, sensory data reveals movement patterns, i.e., walking, running, driving, etc., that occurred during a specific time frame^20^. Thirdly, sensory reading to device associations could be used as corroborative evidence in legal proceedings, strengthening the persecution or defense by demonstrating whether the sensory data aligns with suspect’s actions. Finally, in scenarios that involve data manipulation, this association technique can verify the authenticity and integrity of sensory data, ensuring their admissibility in court. All in all, these capabilities highlight the importance of associating sensory data with its originating device in advancing mobile forensic methodologies.

This paper introduces SENTINEL-DL (SENsory-based Trace IdentificatioN for dEvice anaLysis using Deep Learning), a novel framework for forensic device attribution leveraging motion sensor data. SENTINEL-DL utilizes CNNs to extract deep motion-based signatures from accelerometer readings, enabling precise device attribution with high reliability. By analyzing subtle variations in accelerometer sensor readings, the proposed approach constructs unique digital fingerprints that are resistant to traditional spoofing or tampering techniques. This capability is particularly critical in forensic investigations, where sensor-derived evidence can provide a passive and tamper-resistant mechanism to verify the identity of the device. Empirical evaluations demonstrate the efficacy of SENTINEL-DL in accurately attributing motion sensor data to specific devices. The framework achieves a TPR of 93.99% and 92.65%, with a FAR as low as 0.66% and 1.22%, for smartphones and smartwatches, respectively. Furthermore, SENTINEL-DL attains an overall accuracy of 98.76% for smartphones and 98.97% for smartwatches, highlighting its robustness and reliability in forensic scenarios. These results highlight the potential of accelerometer sensor-based forensic attribution as an alternative to conventional device authentication techniques, reinforcing its application in digital forensic investigations, device fingerprinting, and sensor-based evidence validation. It is important to note that the dataset used in this study provides one physical device per model; therefore, the empirical evaluation focuses on *model-level attribution*. While our broader forensic motivation encompasses both device-level and device-instance attribution, the available data permits us to demonstrate attribution at the granularity of device models.

The key contributions of this paper are as follows.

- The proposal of a passive, non-intrusive forensic framework titled SENTINEL-DL—that leverages accelerometer readings to associate digital evidence with specific smartphone and smartwatch models.
- The demonstration of device-specific acceleration patterns and analysis of intrinsic variations in linear acceleration for fingerprinting and differentiating devices with higher accuracy.
- Evaluation of SENTINEL-DL using state-of-the-art classifiers, i.e., RF, DNN, and CNN. CNN outperformed its coun- terparts, achieving the highest accuracy of 98.76% and 98.97% in distinguishing different smartphone and smartwatch models, respectively.

## Related work

Motion-based data has extensively been used in different domains, such as activity recognition^3,4^, user authentication^7,8^, tracking and behavioral analysis, etc., however, here we consider only studies which have exploited sensory readings for forensic attributions. To the best of our knowledge, no prior work directly addresses 3d motion sensor-based forensic attribution for smartwatch identification, making our approach novel.

The study^21^ explores the efficacy of deep learning architectures such as CNNs, LSTMs, and Transformers for authenticating mobile sensor data; In the context of mobile security, sensor data authentication means confirming the reliability, trustworthiness, and original source of information obtained from sensors to rule out any possibility of data manipulation or falsification. As a result, this study accentuates the need to validate the credibility and provenance of sensor data in mobile security. Our study develops SENTINEL-DL, a framework that applies CNNs to derive motion-based signatures for device identification and forensic attribution using the referenced paper which details the utilization of various deep learning models for sensor data authentication.

The study by Baldini et al.^22^ determines whether it is possible to distinguish between smartphones on the basis of the unique faults evident in Micro Electro Mechanical Systems (MEMS) sensors (e.g., accelerometers and gyroscopes). The authors used a high-precision robotic arm to make controlled and repeatable movements to collect motion sensor data from three identical smartphone models. The extracted statistical features from the sensor data were then used to train a Support Vector Machine (SVM) classifier, which achieved an identification accuracy of over 90%. Similarly, in another study^18^, the authors investigate the possibility of distinguishing between smartphones based on the inherent variability in their in-built sensors. They focus on two primary methods: (1) Speaker-Microphone System Analysis—They capture unique audio signatures specific to each device by examining the frequency response of the speaker and microphone system, and (2) Accelerometer Calibration Errors—They analyse device-specific calibration errors in accelerometers, which are accessible via JavaScript running in a mobile web browser without requiring any permissions or notifying the user.

Our approach aligns with the fundamental concept of utilizing sensor data for device identification but differs in several key aspects: While the study^18^ employed controlled experiments focusing on specific sensors like the speaker-microphone system and accelerometer, our study emphasizes real-world motion sensor data collected during natural user interactions. We employ deep learning models, such as CNNs and DNNs, to extract complex motion signatures (motion signatures refer to distinctive patterns of movement or vibration data that are captured by sensors, such as accelerometers, gyroscopes, or magnetometers. These sensors detect and record physical phenomena like acceleration, rotation, and orientation in three-dimensional space. The collected motion data can be used to identify unique behaviors, activities, or characteristics of a device or its user), enhancing classification accuracy and robustness. Additionally, our work extends beyond smartphones to include smartwatches, demonstrating the applicability of motion sensor-based identification across a broader range of devices. Notably, our approach achieves a TPR of 93.99% and 92.65%, with an overall accuracy exceeding 98% for both smartphones and smartwatches, higher than the state of the art of 90% by^22^ underscoring the effectiveness of our deep learning-based framework in forensic device attribution.

## Our solution—SENTINEL-DL

### Use case

A financial institution, e.g. a bank, discovers unauthorized access to its mobile banking system which has facilitated multiple transactions from user accounts. While traditional authentication logs can show IP addresses and device IDs, these can be easily spoofed or altered^23,24^. Forensic analysts accurately identify the compromised device by analyzing motion sensor readings, i.e., accelerometer readings, of user interactions with the banking app. The smart devices present different sensor responses because of small differences in hardware, usage, and environmental conditions. The investigators can hook the stolen funds to a specific smartwatch after comparing the extracted sensor signature with a database of legitimate user devices that have accessed the system. This method strengthens fraud detection mechanisms by verifying the devices that can access the financial systems, thus improving the overall cybersecurity and forensic accountability.

### Problem definition: forensic attribution of motion sensor data

Given a set of *N* smart devices, each equipped with an accelerometer sensor, the goal is to develop a forensic attribution framework capable of associating motion sensor data with the correct originating device. The fundamental objective is to exploit intrinsic device-specific motion signatures, influenced by manufacturing inconsistencies, calibration differences, and sensor imperfections, to reliably attribute accelerometer recordings to a known device profile.

Formally, we define the forensic attribution problem as follows:

- Let *D* = {*D*_1_*, D*_2_*, ..., D*_*N*_} represent a collection of *N* devices under forensic examination, where each device exhibits distinct motion characteristics inherent to its sensor hardware.
- Each device *D*_*i*_ generates an accelerometer time-series signal *X*_*i*_, defined as:
  1 $$X_{i} = \{ (a_{1} ,a_{2} ,...,a_{T} )\}$$
  where: $a_{t} \in {\mathrm{R}}^{{3}}$ represents the three-dimensional accelerometer readings $\left( {\mathop a\limits_{x}^{t} ,\mathop a\limits_{y}^{t} ,\mathop a\limits_{z }^{t} } \right)$ at time step *t*, capturing linear acceleration along the three spatial axes.

- The objective is to learn a classification function:
  2 $$f:X \to D$$
  that maps an accelerometer time-series signal *X* to its corresponding device label *D*_*i*_, enabling forensic attribution.

- Given an unknown motion sensor recording *X* ^∗^, the trained model predicts the most probable device label $\hat{D}$, satisfying:
  3 $$\hat{D} = \mathop {\arg \max }\limits_{{D_{i} \in D}} P\left( {D_{i} |X^{ * } } \right)$$
  where *P*(*D*_*i*_|*X* ^∗^) denotes the probability that the motion sensor signature belongs to device *D*_*i*_.

This formulation provides a foundation for forensic investigations where sensor-based device attribution is essential for verifying the authenticity of digital evidence. The learned model effectively distinguishes devices based on their motion signatures, facilitating robust forensic analyses in scenarios where traditional authentication methods may be compromised. Given the constraints of the HHAR dataset, which contains one physical device per model, the attribution task in this study is performed at the *model level*. That is, the classifier distinguishes among different smartphone and smartwatch models rather than between multiple physical instances of the same model. We explicitly acknowledge this distinction to avoid ambiguity between device-instance attribution and model-level attribution.

### Our approach

**Fig. 1:**
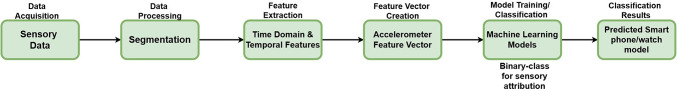
Overview of the methodology for forensic attribution of smartphone and smartwatch motion sensor data. The pipeline consists of data preprocessing, feature extraction, classifier training, attribution analysis, and evaluation on unseen data to ensure accurate and reliable forensic attribution.

The approach used in this study follows a structured pipeline of methods for processing and analyzing accelerometer sensor data for forensic attribution of motion sensor recordings, as shown in Fig. 1. The process starts with data preprocessing, where accelerometer data sampled at approximately 160 Hz is divided into fixed-length windows of 200 samples approximately equal to 1.25 seconds of motion data. Applying uniform segmentation to the dataset helps extract meaningful statistical features from motion signals while standardizing the data. After preprocessing, the raw sensor data is transformed into structured representations that contain unique motion signatures through feature extraction. For every window of data, statistical features such as mean, variance, skewness, kurtosis, and mode are thoroughly calculated across all three axes. The motion magnitude is calculated to obtain aggregate movement characteristics. The extracted features are used for forensic attribution, transforming time-series data into structured feature vectors that allow for classification.

In the next stage, extracted feature vectors are used to train machine learning models optimized for forensic attribution. The classifiers, RF, DNN, and CNN are designed to correlate motion sensor data with device models. The classifiers are the backbone of the forensic attribution framework, which allows the precise association of motion sensor recordings with their source devices. Finally, the models are evaluated on unseen data to ensure that they generalize well and are robust in real-world forensic scenarios. This structured pipeline is followed by the study to create a scalable, interpretable, and computationally efficient approach to motion sensor data attribution. This methodology not only helps in the correct identification of devices for forensic purposes but also enhances the reliability of motion sensor data which makes it more useful for digital investigations and forensic analysis.

## Methodology

In this section, we explain our applied methodology.

### Dataset

The dataset used in this study is the *Heterogeneity Dataset for Human Activity Recognition from Smartphones and Smartwatches*, designed to benchmark human activity recognition algorithms such as classification, automatic data segmentation, sensor fusion, and feature extraction. The dataset addresses challenges arising from sensor heterogeneities and is publicly available^25^. The dataset contains motion sensor data collected from smartphones and smartwatches while users performed different activities, recorded in no specific order. The activities included in the dataset are *biking*, *sitting*, *standing*, *walking*, *stair up*, and *stair down*. Although the HHAR dataset provides both accelerometer and gyroscope measurements, SENTINEL-DL uses only the accelerometer data in this study. All feature extraction, segmentation, and classification steps are based solely on the 3-axis accelerometer signal. Any gyroscope fields in the dataset are not used in our forensic attribution pipeline.

The dataset comprises of four files, corresponding to readings from smartphones and smartwatches, and further divided by sensor type: *Phones accelerometer.csv*, *Phones gyroscope.csv*, *Watch accelerometer.csv*, and *Watch gyroscope.csv*. Each file contains time-series samples represented as rows with columns that capture the following information: the *Index* (row number), *Arrival Time* (timestamp when the measurement reached the sensing application), *Creation Time* (timestamp assigned to the sample by the operating system), and the *x*, *y*, *z* values from the sensors. Additional columns include *User*, indicating the user from whom the sample was collected (labeled *a* through *i*), *Model*, specifying the phone or smartwatch model, *Device*, providing the specific device identifier (e.g., *nexus4 1*, *s3mini 2*), and *gt*, representing the ground truth activity label (*bike*, *sit*, *stand*, *walk*, *stairsup*, *stairsdown*, or *null* for undefined activity). We exploit *Phones accelerometer.csv* and *Watch accelerometer.csv* files in this study.

**Fig. 2:**
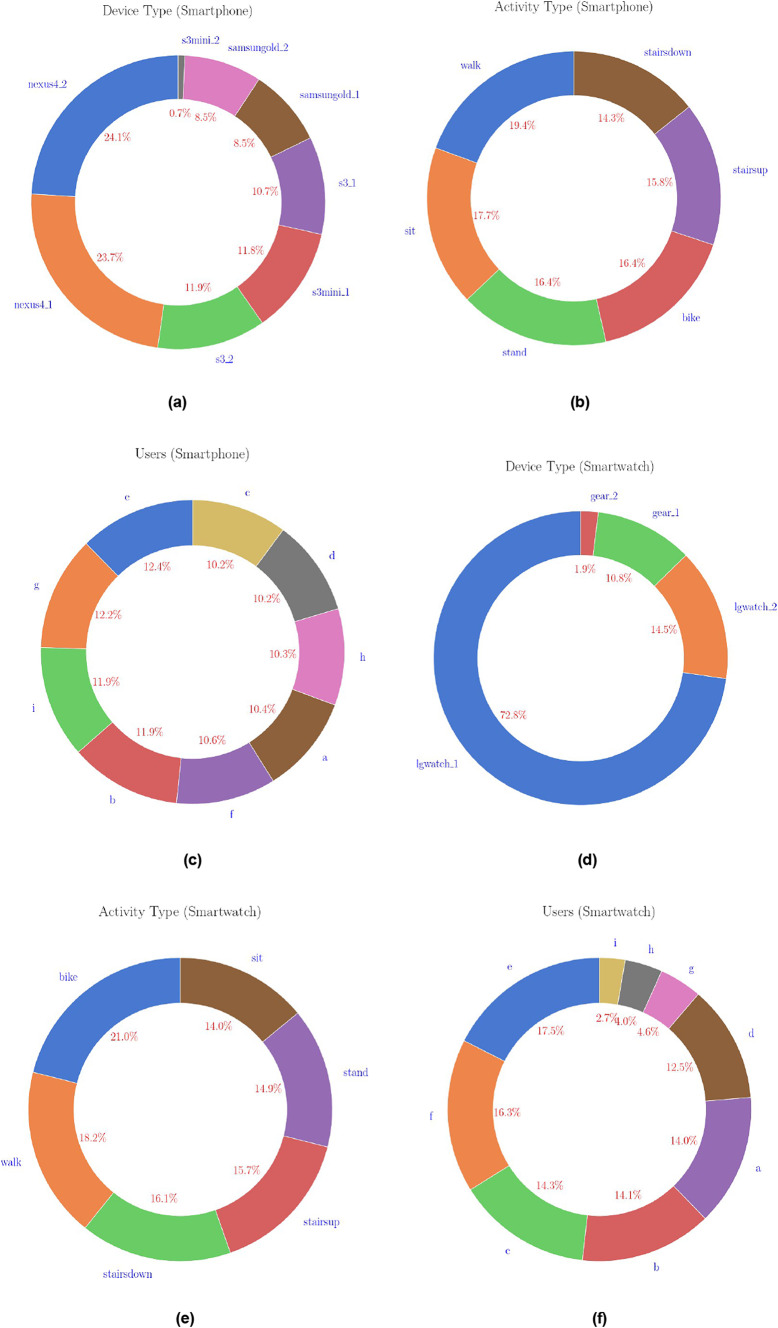
Distribution of samples across device types, activity types, and users.

The dataset includes data from eight smartphones (see Fig. 2a) and four smartwatches (see Fig. 2d). The smartphones used were two Samsung Galaxy S3, two Samsung Galaxy S3 mini, two LG Nexus 4, and two Samsung Galaxy S+. The smartwatches used were two LG G Watches and two Samsung Galaxy Gears. Nine users, identified as *a* through *i* (see Fig. 2c and f), participated in the data collection process, performing the activities under varying conditions (see Fig. 2b and e). The dataset is consistent across files in terms of user identifiers and device naming conventions. However, there are some inconsistencies in the number of samples for specific activities due to sampling issues. For example, User *h* has fewer samples for the *sitting* activity in the *Phones accelerometer.csv* file.

This dataset has been utilized in prior research to address challenges in mobile sensing and human activity recognition. For further details on the dataset and its collection process, readers are referred to the publication by Stisen et al.^26^.

The heterogeneity dataset for human activity recognition from smartphones and smartwatches dataset provides one device instance per model; therefore, our experiments reflect model-level differentiation. Although the forensic motivation includes device-instance attribution, this level of granularity is not supported by the available dataset, and we make this distinction explicit in the revised manuscript. Furthermore, the HHAR dataset provides two physical devices for each smartphone and smartwatch model. In this study, these two units were not treated as separate attribution targets; instead, their samples were combined and labeled under the same device-model class (e.g., both Nexus 4 units form a single class). During dataset partitioning, samples from both physical units were included in the stratified random split, with 67% used for training and 33% for testing. Consequently, the evaluation reflects model-level attribution rather than distinguishing between individual physical instances of the same model.

### Dataset preprocessing

The raw dataset, collected from smartphones and smartwatches, was sampled at the highest possible frequency supported by the respective devices. However, the exact sampling frequency is not explicitly stated in the original paper or accompanying documentation. To address this, we computed the approximate sampling frequencies using the timestamp information provided in the dataset. Based on this analysis, we identified an average sampling frequency of approximately 160 Hz for smartphone accelerometer data and 43.26 Hz for smartwatch accelerometer data.

To prepare the dataset for analysis, we segmented the time-series data into fixed-length windows of 200 samples each, corresponding to approximately 1.25 seconds of data per segment. This segmentation was chosen to balance temporal resolution with computational efficiency, ensuring that the segments were long enough to capture meaningful activity patterns while remaining computationally manageable. We examined whether an alternative public dataset could be used to evaluate cross-dataset generalization. However, current motion-sensor datasets such as WISDM, MobiAct, MotionSense, RealWorld HAR, and UCI HAR do not include device identifiers or multiple physical units of the same model, which are essential for device attribution. Consequently, no comparable public dataset exists for validating device-level forensic attribution across datasets.

### Feature extraction

The segmentation process provided a consistent structure to the data, enabling a standardized approach for feature extraction and subsequent model training. Each segment, corresponding to a 1.25-second window of data, served as a foundation for calculating statistical features. We believed, a 1.25-second window captures sufficient sensor dynamics to uniquely characterize device-specific motion patterns while remaining short enough to ensure forensic relevance and temporal precision.

For each segment, we computed a comprehensive set of statistical features from the accelerometer readings along the *x*, *y*, and *z* axes. Additionally, we calculated the magnitude scalar (*ms*), which represents the overall motion intensity^7^ and is derived as:

$$ms = \surd x^{2} + y^{2} + z^{2} .$$

The following statistical features were extracted for each axis (*x*, *y*, *z*) and the magnitude scalar (*ms*) to comprehensively characterize motion sensor data:

- Mean: The average value of the sensor readings, providing a measure of central tendency.
- Median: The middle value of the sensor readings, offering robustness against outliers.
- Variance: A measure of the variability in the sensor readings, indicating the spread of the data.
- Standard deviation: The square root of the variance, reflecting the dispersion of values around the mean.
- Skewness: A measure of the asymmetry in the distribution of the sensor readings.
- Kurtosis: A measure of the ”tailedness” of the distribution, indicating the presence of outliers.
- Mode: The most frequently occurring value in the segment, capturing dominant patterns in the data.
- Energy: The sum of squared values within a segment, representing the overall signal power and intensity.
- Zero crossings: The number of times the signal crosses the zero threshold, capturing oscillatory behavior and frequency patterns.

By computing these features for each of the four dimensions (*x*, *y*, *z*, and *ms*), we created an extensive feature vector for each segment. Specifically, nine statistical features were calculated per dimension, resulting in a total of 36 features per segment. This enriched feature representation captures crucial aspects of motion sensor data, including central tendency, variability, frequency domain characteristics, and dominant signal patterns. Each 1.25-second window is treated as an independent sample, and the 36 extracted features form a single feature vector representing that segment. All segments are subsequently aggregated into the dataset as separate, unordered instances. Temporal continuity between adjacent segments is not preserved, as the objective of forensic attribution is to capture device-specific intrinsic motion signatures rather than model sequential behavioral patterns. This choice reflects the fact that hardware-induced sensor characteristics (e.g., calibration biases and manufacturing tolerances) manifest consistently within individual windows, enabling reliable attribution without requiring temporal dependencies across segments.

### Classifier selection and parameter optimization

Classifiers serve as fundamental components in machine learning, enabling models to learn patterns from data and make informed decisions by assigning labels to new, unseen samples. In this study, we employed RF, DNN, and CNN as our primary classification models. These choices were informed by their demonstrated effectiveness in prior research^27–30^, particularly in domains requiring high accuracy and robust feature learning.

To maximize the performance of these classifiers, we conducted an extensive parameter optimization process, fine-tuning key hyperparameters to enhance predictive accuracy and generalization. Parameter optimization plays a crucial role in refining model behavior by identifying the most effective configurations that improve classification performance. A variety of optimization techniques exist, including grid search, random search, cross-validation, Bayesian optimization, evolutionary algorithms, and meta-learning, each offering distinct advantages in balancing computational efficiency with model accuracy.

Our optimization strategy systematically explored a range of hyperparameter settings, as detailed in Table 1, ensuring that the most effective configurations were selected for the final model deployment. It is worth noting that we applied RF in its default settings.

**Table 1:** Parameter optimization of all chosen classifiers.

Classifiers	Parameters and their range		Best	Best validation accuracy (%)
	num_layers	{2,…, 10}	2	
DNN	num_units learning_rate	{32*i* | 1 ≤ *i* ≤ 16} {10^−*i*^ | 2 ≤ *i* ≤ 4}	416, 320 10 − 3	96.25
	filters	{32, 64, 128, 160, 192, 224, 256}	224	
CNN	kernel_size dense_units	{3, 5} {32*i* | 1 ≤ *i* ≤ 10}	3 256	100
	dropout_rate	{0.1*i* | 1 ≤ *i* ≤ 5}	0.2	

It is important to note that the all the classifiers in SENTINEL-DL do not operate on raw accelerometer time-series data. Instead, each 200-sample window is transformed into a 36-dimensional statistical feature vector, and they are trained directly on these feature vectors. Thus, the CNN functions as a feature-based deep classifier rather than a temporal convolutional model.

### Classification protocol

This study is centered on forensic device attribution, where the objective is to determine whether a given motion sensor recording originates from a specific smartphone or smartwatch model. The problem is formulated as a binary classification task, where sensor data is categorized into two distinct classes:

- Original: Motion sensor data collected from the specific device model under investigation.
- Non-original: Motion sensor data collected from all other device models.

The primary goal is to develop a classification framework capable of accurately attributing motion-based sensor data to its originating device. By analyzing accelerometer readings, the study investigates whether inherent motion signatures are sufficiently distinct to enable reliable forensic attribution. This approach is particularly relevant in forensic investigations where establishing a direct link between sensor data and a device is crucial for digital evidence validation. Because the HHAR dataset provides only one physical device per model, the attribution task is performed at the *device-model level*. In this context, “original” refers to samples originating from the target device model, whereas “non-original” denotes samples from all other models. Device-instance attribution (i.e., distinguishing between multiple physical units of the same model) cannot be evaluated with this dataset, and our terminology has been updated to reflect this distinction.

To train and evaluate the attribution models, we applied a stratified random split of the dataset, ensuring that both the *original* and *non-original* classes were proportionally represented in each subset. Consistent with the state-of-the-art work, 67% of the samples were used for training and the remaining 33% were set aside as a held-out test set. This explicit split ensures class balance and prevents sampling bias during evaluation. This splitting also ensures that the trained models generalize well to unseen data, thereby enhancing their forensic applicability. The attribution models, including RF, DNN, and CNN, are systematically trained and optimized to maximize attribution accuracy while minimizing misclassification errors.

Forensic attribution experiments are conducted by training each model on labeled accelerometer recordings, ensuring a balanced representation of *original* and *non-original* samples. The classification function then evaluates whether an unknown motion sensor segment belongs to the target device model or originates from a different device. By structuring the problem in this manner, the study provides a rigorous framework for forensic device attribution, reinforcing the role of motion sensor data as a credible source of digital evidence.

## Results and discussion

### Training setup and computational resources

All models used in this study (RF, DNN, and CNN) were trained from scratch without any pre-trained weights or transfer learning. Training was conducted on a standard workstation equipped with an NVIDIA GTX-series GPU (8–12 GB VRAM), 32 GB of RAM, and an Intel i7-class processor. Due to the compact size of the feature vectors and moderate model complexity, the computational requirements are modest.

On this hardware, training the RF model completed within seconds, while the DNN and CNN required approximately 2–5 minutes per device-attribution experiment. These durations include hyperparameter tuning and model optimization. The relatively low computational demand demonstrates that SENTINEL-DL is lightweight and feasible to deploy in forensic laboratory environments without requiring specialized high-performance computing resources.

### Success metric

We assess the performance of our models for device attribution using key forensic evaluation metrics that measure classification accuracy, reliability, and discrimination capability. Specifically, we report TPR, FNR, FPR, TNR, Accuracy, F1 Score, and Area Under the Curve (AUC).

In the forensic attribution process, the dataset is structured into two classes: original, representing sensor data collected from the target device, and non-original, representing sensor data from all other devices. The TPR measures the proportion of correctly attributed device instances, ensuring that motion sensor data is accurately linked to its originating device. Conversely, FPR quantifies the proportion of misclassified non-original samples, which is critical in forensic investigations where minimizing false attributions is essential. Since *FNR* = 1–*TPR* and *TNR* = 1–*FPR*, we primarily report TPR and FPR to avoid redundancy.

Additionally, accuracy provides an overall measure of correct attributions, while the F1 Score captures the balance between precision and recall, particularly in cases where dataset imbalances may impact forensic decision-making. The Receiver Operating Characteristic (ROC) Curve further illustrates the trade-off between TPR and FPR across different classification thresholds, with AUC quantifying the model’s ability to distinguish between original and non-original devices. A high AUC value reinforces the forensic reliability of device attribution by ensuring precise differentiation, thereby strengthening the evidentiary value of motion sensor data in forensic investigations.

## Results

**Fig. 3:**
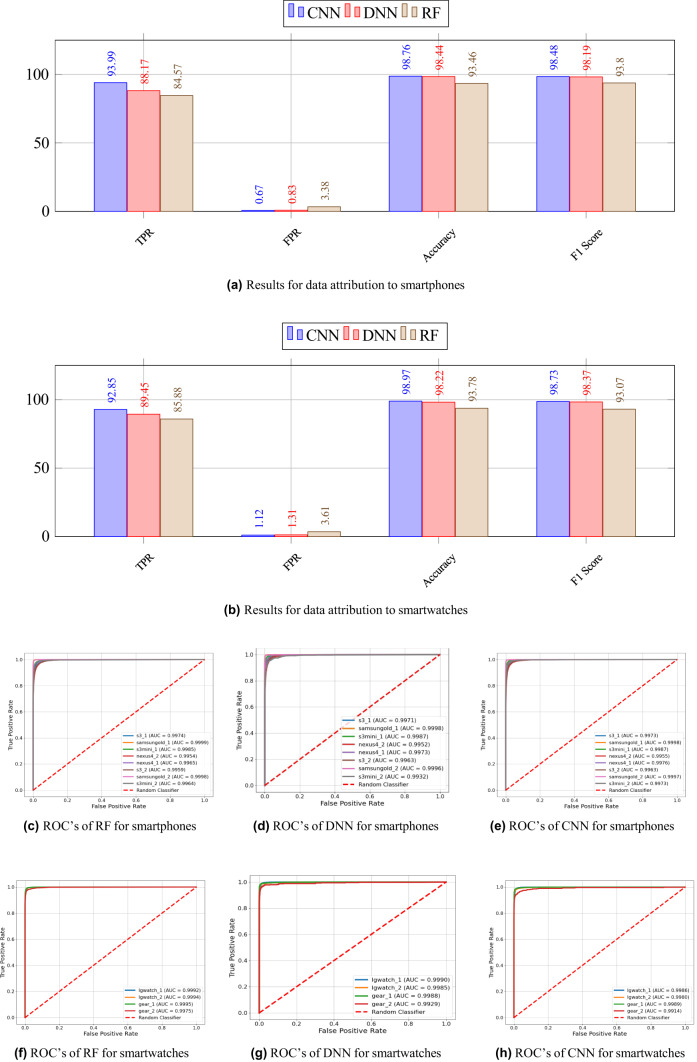
Accuracy of device attribution (in %) of (**a**) smartphones, and (**b**) smartwatches. We show the ROC curves for different classifiers for smartphones (**c**), (**d**), and (**e**) and for smartwatches (**f**), (**g**), and (**h**).

We summarize our obtained results in Fig. 3.

The results presented in Fig. 3a and b illustrate the performance of three classification models— CNN, DNN, and RF— for the task of sensory data attribution to smartphones and smartwatches, respectively.

In the context of smartphone data attribution, as depicted in Fig. 3a, CNN achieves the highest TPR of 93.89%, indicating its superior ability to correctly identify the original device data. This is followed by DNN and RF, which attain TPRs of 91.87% and 84.57%, respectively. A lower FPR is critical in forensic applications, as it ensures that non-original devices are not falsely classified as the original device. CNN shows the lowest FPR at 0.67%, while DNN and RF have slightly higher FPR values of 0.88% and 3.38%, respectively. The accuracy results further highlight the robustness of CNN, which achieves an accuracy of 98.76%, marginally outperforming DNN (98.45%) and significantly surpassing RF (93.46%). Similarly, the F1 Scores, which balance precision and recall, follow the same trend, with CNN achieving 98.48%, DNN attaining 98.19%, and RF scoring the lowest at 93.80%. These results demonstrate that CNN is the most effective model for smartphone data attribution, offering the highest reliability with minimal mis-classification errors.

A similar performance pattern is observed for smartwatch data attribution in Fig. 3b. CNN again exhibits the highest TPR of 92.85%, compared to 89.45% for DNN and 85.88% for RF. Notably, the FPR values are slightly higher than those observed in the smartphone attribution task, with CNN showing 1.12%, DNN at 1.31%, and RF at 3.61%. Despite these minor increases, CNN continues to outperform the other models, achieving an accuracy of 98.97%, while DNN and RF achieve 98.22% and 93.78%, respectively. The F1 Score follows the same ranking, with CNN scoring 98.73%, followed by DNN at 98.37% and RF at 93.07%. These findings indicate that CNN maintains its superior performance across different device types, reinforcing its capability to accurately attribute sensor data with minimal errors.

The ROC curves presented in Fig. 3 also provide further insight into the classification performance of the RF, DNN, and CNN models for device attribution to smartphones and smartwatches. These curves illustrate the trade-off between the TPR and FPR at various classification thresholds, offering a visual representation of each model’s ability to distinguish between original and non-original device data.

For smartphone attribution, the ROC curves in Fig. 3c, d, and e correspond to the performance of RF, DNN, and CNN, respectively. All the models, especially, CNN exhibits the highest AUC, indicating its superior discriminatory power in correctly attributing device data. The RF model, shows comparatively lower AUC values, signifying a higher likelihood of misclassification in certain cases. The sharp rise in the TPR at low FPR values in CNN and DNN curves highlights their robustness in correctly identifying device attributions with minimal false positives. The DNN model, while still effective, demonstrating strong classification capabilities with an AUC slightly lower than that of CNN.

Similarly, Fig. 3f illustrate the ROC curves for smartwatch attribution using RF, DNN, and CNN, respectively. The patterns observed in smartphone attribution hold true for smartwatch attribution as well, with all the models achieving the highest AUC, exhibiting supreme performance. The ROC curves of all the classifiers remain tightly clustered near the top-left corner, highlight their ability to effectively separate original and non-original devices.

Deep learning models, particularly CNNs, are also susceptible to overfitting, where the model learns patterns specific to the training data but fails to generalize effectively to unseen cases. Overfitting can compromise the forensic reliability of device attribution by introducing classification inconsistencies when applied to new sensor data. To ensure that our models maintain generalization capability, we conducted an overfitting analysis by monitoring accuracy and loss curves during training and validation.

**Fig. 4:**
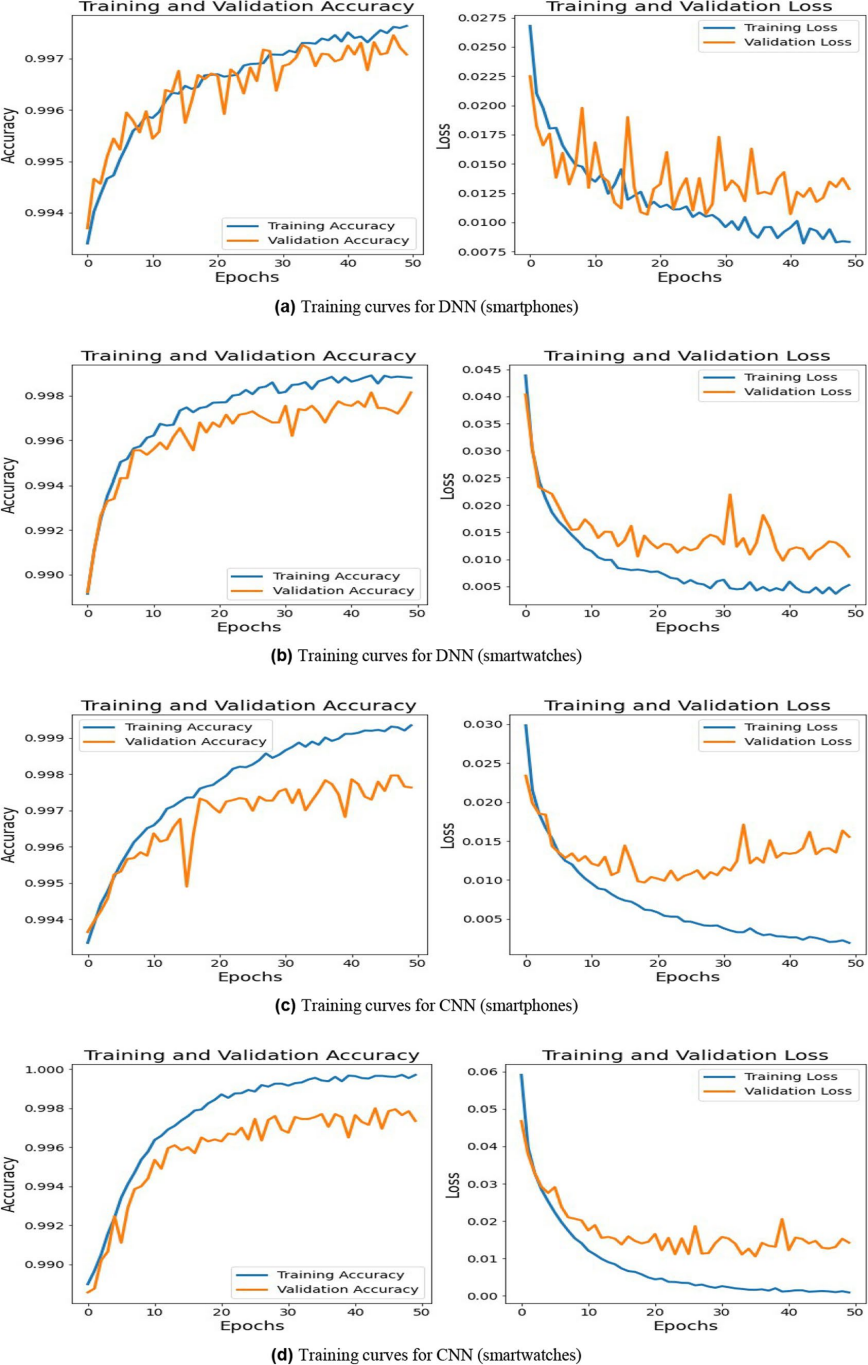
Training curves of DNN and CNN classifiers for sensory reading attribution to smartphones and smartwatches. *Note* Each subplot is intentionally zoomed to show the relevant accuracy/loss range for that model, as uniform scaling would obscure the visible convergence patterns.

As depicted in Fig. 4, the training and validation accuracy curves remain closely aligned throughout the learning process, while the loss curves exhibit a steady decline without significant divergence. This indicates that the classifiers generalize well across different motion sensor recordings and do not exhibit signs of overfitting. The stability of these curves confirms that SENTINEL-DL maintains high attribution accuracy across both training and unseen data, highlighting its applicability in real-world forensic scenarios.

In general, these results highlight the effectiveness of deep learning models, particularly CNN, in the forensic attribution of motion sensor data to specific devices. CNN consistently demonstrates the highest classification accuracy, lowest false attribution errors, and superior balance between TPR and FPR. The performance of DNN is comparable, though slightly lower, while RF lags significantly behind in all metrics. These results highlight the potential of deep learning approaches in forensic investigations, where precise and reliable device attribution is critical for ensuring the integrity and validity of digital evidence. It is important to emphasize that SENTINEL-DL is intended as a supporting or corroborative forensic technique rather than a stand-alone determinant of device identity. Motion-sensor–based attribution provides probabilistic evidence based on sensor calibration biases and hardware-induced signatures, and is most valuable when used to reinforce or cross-validate other digital artefacts such as device logs, network records, account activity, or physical evidence recovered during an investigation. As in most forensic workflows, the method is designed to complement—not replace—established attribution mechanisms, particularly in scenarios where conventional identifiers may be absent, spoofed, or unreliable.

### Limitations

While SENTINEL-DL demonstrates strong potential for forensic device attribution, several limitations however exist. Firstly, the evaluation relies on a single public dataset that, although heterogeneous, does not include a wider variety of device families or multiple units from newer models. The absence of comparable publicly available datasets with device identifiers limits large-scale cross-dataset generalization. Secondly, the data was collected under semi-controlled activity scenarios, which may not fully reflect the variability of motion encountered during spontaneous real-world interactions. Thirdly, although our models capture intrinsic hardware-induced sensor characteristics, subtle variations in user behavior or environmental factors may introduce noise that affects attribution performance. Finally, this study does not include adversarial testing, such as sensor spoofing or intentional manipulation of motion data, which represents an important area for future forensic robustness evaluations. All in all, these limitations highlight opportunities for future research, including the creation of richer device-labeled datasets, collection of fully uncontrolled real-world motion data, and the study of adversarial resilience in sensor-based attribution systems. Another important aspect is the interpretability of the attribution model. Although this work focuses on establishing feasibility and measuring attribution accuracy, explainable AI (XAI) techniques, e.g., SHAP, permutation feature importance, or gradient-based attribution, could provide additional insight into which statistical motion features most strongly influence device-level decisions. Integrating these methods could represent a valuable direction for future work, particularly for strengthening the forensic interpretability and transparency of motion sensor–based attribution systems.

Although the attribution task is designed to capture device-specific motion signatures, we acknowledge that the HHAR dataset contains variability introduced by user movement styles and activity types. Several elements of our methodology, i.e., short windowing, stratified sampling across all users and activities, and the use of statistical features linked to sensor calibration and noise, helped reduce the influence of user- or activity-specific patterns. As each device model is used by multiple users across diverse activities, the classifier receives heterogeneous behavioral input that limits overfitting to a single user or activity. Nevertheless, a small degree of behavioral influence cannot be entirely excluded, and future work may include strictly user-independent validation or controlled multi-instance datasets to further isolate device-only signatures.

We also note that a practical forensic requirement is the ability to generalize attribution to previously unseen sessions or unseen users operating the same device model. The HHAR dataset does not contain multiple sessions per user or repeated recordings from the same user–device pair over time, which limits the ability to perform a strictly user-independent or session- independent evaluation. While our use of multiple users per model and short, feature-based windows reduces overfitting to individual behavioral patterns, we acknowledge that full generalization to unseen sessions cannot be tested with the present data. Future work will incorporate datasets with repeated sessions, multiple physical instances per model, and longitudinal recordings to more rigorously validate session- and user-independent forensic attribution.

It is important to note that the current study does not assume an active adversary capable of manipulating the motion-sensor pipeline. Our attribution framework operates on unaltered accelerometer readings as provided by the dataset and does not consider replay attacks, synthetic trace injection, driver-level manipulation, or app-level interception of sensor streams. While SENTINEL-DL leverages hardware-induced signatures that are inherently difficult to spoof, a comprehensive adversarial threat model and an evaluation of robustness against sensor-level attacks remain important directions for future work.

Although the HHAR dataset includes a diverse set of daily activities, our evaluation does not explicitly measure attribution robustness across individual activity types, carry positions, or environmental conditions. The classifier is trained on a heterogeneous mix of all recorded activities, which encourages learning of device-specific sensor characteristics rather than activity-dependent motion patterns. The short segmentation window (1.25 s) and feature set emphasize statistical properties of the sensors that are less sensitive to behavioral context. Nevertheless, real-world forensic scenarios may involve variations in carry position, gait, or environmental motion not represented in the dataset. Evaluating activity-independent and environment- independent robustness remains an important direction for future work.

## Conclusions

This study introduces SENTINEL-DL, a deep learning-based forensic attribution framework that leverages accelerometer sensor readings to establish a robust, tamper-resistant mechanism for device attribution. By analyzing the unique motion signatures captured by built-in accelerometers, the proposed approach enables precise differentiation between smartphone and smartwatch models.

Our experimental evaluation demonstrated the effectiveness of SENTINEL-DL in accurately attributing motion sensor data to specific devices. Among the tested classifiers—RF, DNN, and CNN–CNN achieved the highest attribution accuracy, with a TPR of 93.99% for smartphones and 92.65% for smartwatches, alongside a FAR of 0.66% and 1.22%, respectively. Additionally, the overall accuracy reached 98.76% and 98.97%, reinforcing the forensic reliability of motion sensor-based attribution. The ROC curves and high AUC values further confirm the model’s strong discriminatory power in distinguishing between *original* and *non-original* device attributions. A critical concern in deep learning models, particularly CNNs, is their vulnerability to overfitting, which could impact generalizability. To address this, the training and validation accuracy/loss curves were analyzed (Fig. 4), revealing no signs of overfitting. The model maintained stable learning behavior, indicating that it generalizes well to unseen forensic data. This aspect strengthens the framework’s applicability in real-world forensic scenarios, ensuring that its attribution capabilities remain reliable across diverse motion sensor recordings.

These findings highlight the viability of motion sensor-based device attribution as a passive, non-intrusive, and lightweight forensic tool. Unlike traditional device authentication methods, which can be circumvented through MAC address spoofing, cryptographic key extraction, or hardware tampering, SENTINEL-DL provides an additional forensic layer by utilizing inherent device-specific motion signatures. Looking ahead, future research could explore the integration of additional motion-based modalities, such as gyroscope readings or magnetometer data, to further enhance attribution robustness. Additionally, expanding the dataset to include a wider variety of device models and testing attribution across different environmental conditions could improve the generalizability of the proposed approach. Finally, further adversarial testing could be conducted to assess the resilience of motion sensor-based forensic attribution against sophisticated attacks, such as sensor spoofing or adversarial perturbations.

## Data Availability

The dataset *Heterogeneity Dataset for Human Activity Recognition from Smartphones and Smartwatches* used in this study is publicly available^25^. However, the processed version of the data used for training and evaluation in the current research is available from the corresponding author upon reasonable request.
